# 4F-Indole Enhances the Susceptibility of Pseudomonas aeruginosa to Aminoglycoside Antibiotics

**DOI:** 10.1128/spectrum.04519-22

**Published:** 2023-03-28

**Authors:** Qin Dou, Yuxiang Zhu, Chunhui Li, Zeran Bian, Huihui Song, Ruizhen Zhang, Yingsong Wang, Xile Zhang, Yan Wang

**Affiliations:** a College of Marine Life Sciences, and Institute of Evolution & Marine Biodiversity, Ocean University of China, Qingdao, China; The University of North Carolina at Chapel Hill

**Keywords:** halogenated indoles, antibiotic resistance, *Pseudomonas aeruginosa*, aminoglycoside antibiotics, virulence factors

## Abstract

Infections caused by multidrug-resistant bacteria are becoming increasingly serious. The aminoglycoside antibiotics have been widely used to treat severe Gram-negative bacterial infections. Here, we reported that a class of small molecules, namely, halogenated indoles, can resensitize Pseudomonas aeruginosa PAO1 to aminoglycoside antibiotics such as gentamicin, kanamycin, tobramycin, amikacin, neomycin, ribosomalin sulfate, and cisomicin. We selected 4F-indole as a representative of halogenated indoles to investigate its mechanism and found that the two-component system (TCS) PmrA/PmrB inhibited the expression of multidrug efflux pump MexXY-OprM, allowing kanamycin to act intracellularly. Moreover, 4F-indole inhibited the biosynthesis of several virulence factors, such as pyocyanin, type III secretion system (T3SS), and type VI secretion system (T6SS) exported effectors, and reduced the swimming and twitching motility by suppressing the expression of flagella and type IV pili. This study suggests that the combination of 4F-indole and kanamycin can be more effective against P. aeruginosa PAO1 and affect its multiple physiological activities, providing a novel insight into the reactivation of aminoglycoside antibiotics.

**IMPORTANCE** Infections caused by Pseudomonas aeruginosa have become a major public health crisis. Its resistance to existing antibiotics causes clinical infections that are hard to cure. In this study, we found that halogenated indoles in combination with aminoglycoside antibiotics could be more effective than antibiotics alone against P. aeruginosa PAO1 and preliminarily revealed the mechanism of the 4F-indole-induced regulatory effect. Moreover, the regulatory effect of 4F-indole on different physiological behaviors of P. aeruginosa PAO1 was analyzed by combined transcriptomics and metabolomics. We explain that 4F-indole has potential as a novel antibiotic adjuvant, thus slowing down the further development of bacterial resistance.

## INTRODUCTION

Pseudomonas aeruginosa is a prevalent multidrug-resistant pathogen in hospital-acquired infections that readily enters the host and causes infection when the host is immunodeficient or has a compromised barrier (1, 2). Aminoglycosides are commonly used in the treatment of P. aeruginosa infections (3). However, the problem of resistance has become a widespread concern, and bacteria resistant to antibiotics (such as kanamycin, gentamicin, tobramycin, and amikacin) exist in almost every region (4). The resistance mechanisms of P. aeruginosa against antibiotics are complex, among which those associated with resistance to aminoglycoside antibiotics are mainly restriction of inner membrane transport, active efflux pumps, enzymatic inactivation, and mutation in ribosomal proteins (5, 6). One of the most efficient resistance mechanisms is the overexpression of efflux pumps of the resistance-nodulation-cell division (RND) family (7, 8). Notably, MexXY-OprM is an exclusive efflux pump for aminoglycoside antibiotics transportation (9). MexZ, a suppressor of MexXY, is regulated by ArmZ. P. aeruginosa senses when antibiotic-mediated ribosome disruption induces PA5471 (ArmZ) upregulation and interferes with the DNA-binding activity of MexZ, thus activating the efflux pump to increase antibiotic resistance (10). Beyond bacterial resistance against antibiotic, the production of multiple virulence factors, such as pyocyanin and exotoxins, plays a key role in infections caused by P. aeruginosa. The flagella and type IV pili in P. aeruginosa dominate swimming and twitching motility, initiating surface attachment of microcolonies and resulting in activating biofilm formation, which protects cells from the host immune system and antibiotics (11–13).

The two-component system (TCS) is composed of a histidine kinase (HK) and a cytoplasmic response regulator (RR) that senses and responds to external signals, and some TCSs contribute to bacterial virulence and survival (14, 15). In P. aeruginosa, 64 histidine sensor kinases and 72 responses have been identified (16). TCS PmrA/PmrB consisting of PmrB (HK) and PmrA (RR) was reported to modulate the resistance of P. aeruginosa to polymyxin B by modifying lipopolysaccharide (LPS) (17). Acidic pH and acidification of eDNA constitute a signal that could be sensed by P. aeruginosa to induce PhoP/PhoQ and PmrA/PmrB to regulate aminoglycoside resistance by amidoarabinose modification of lipid A and production of spermidine, which may lead to reduced antibiotic uptake (18).

Indole is the basic structure of many natural products and drugs, and many indole derivatives have been used in the clinical treatment of diseases, such as vinblastine, zolmitriptan, and reserpine (19, 20). Indole acts as a signaling molecule that regulates the resistance and tolerance of indole-producing and non-indole-producing bacteria and affects the formation of biofilm and persister cells (21). Researchers have designed a series of indole compounds using the indole as scaffolds to work with antibiotics (22–24). Halogens are a kind of element in the small molecule drug library that can increase the lipophilicity of molecules and improve the permeability to lipid membranes, and their electronegativity can improve the biological activity of drugs (25, 26). Halogenated indoles include 5-iodoindole (5I-indole), 4-fluoroindole (4F-indole), 7-chloroindole (7Cl-indole), and 7-bromoindole (7Br-indole), which reduce the antibiotic resistance of Escherichia coli and Staphylococcus aureus by eliminating persister cells and inhibiting biofilm formation (27). The 7-fluoroindole (7F-indole) inhibits P. aeruginosa PAO1 infection by regulating the production of bacterial virulence (such as pyocyanin and rhamnolipids) and biofilm formation; however, it does not inhibit the growth of planktonic cells (28). Some indole derivatives inhibit antibiotic resistance in bacteria and have antivirulence properties against fungal pathogens. The 4F-indole, 5-fluoroindole (5F-indole), and 7F-indole significantly inhibit the growth of mycelial in Botrytis cinerea (29). Although some halogenated indoles have been reported to be toxic to certain eukaryotic cells (25, 30), the function of nontoxic halogenated indoles and the mechanism by which they regulate bacterial resistance remain unclear. These studies will provide a reliable theoretical basis for the subsequent application of halogenated indoles.

In this study, we showed that 4F-indole enhanced the susceptibility of P. aeruginosa PAO1 to aminoglycoside antibiotics through the possible inhibition of MexXY-OprM efflux pump expression. We further found that this 4F-indole-induced response is regulated by PmrA/PmrB. Moreover, 4F-indole inhibits virulence factors biosynthesis and motility in P. aeruginosa PAO1. Moreover, 4F-indole enhanced central carbon metabolism. This study provides a biochemical and metabolic theory for the reactivation of aminoglycoside antibiotics to treat infections caused by P. aeruginosa PAO1.

## RESULTS

### Halogenated indoles have synergistic effects with antibiotics.

**FIG 1:**
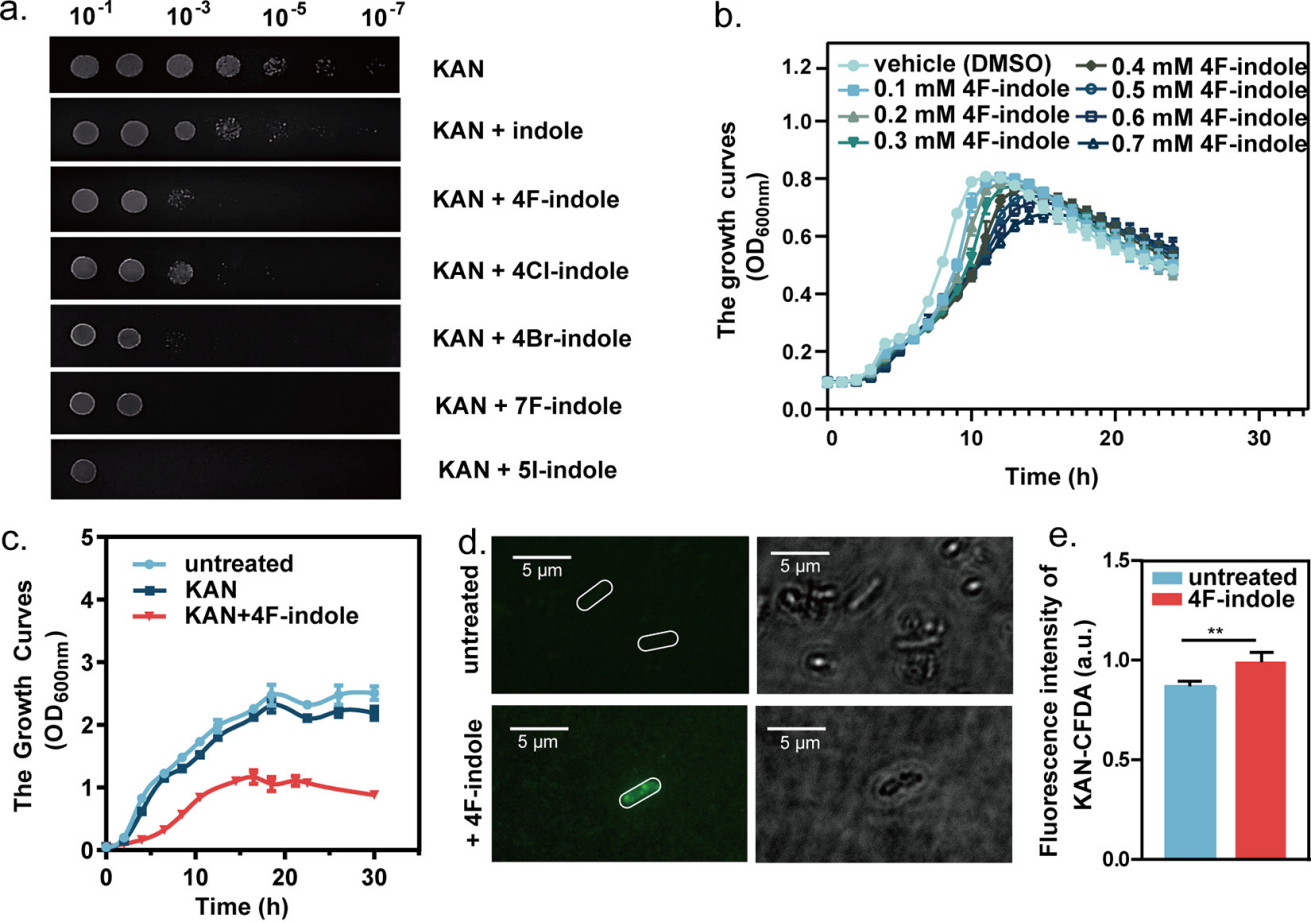
4F-Indole and other halogenated indoles inhibit the growth of P. aeruginosa PAO1 by synergizing with antibiotics. (a) Efficiencies of colony formation of P. aeruginosa PAO1 in the presence of halogenated indoles and kanamycin (KAN). (b) Growth curves of P. aeruginosa PAO1 under different concentrations of 4F-indole treatment. (c) growth curves of P. aeruginosa PAO1 that treated by 4F-indole (0.5 mM) combined with kanamycin (50 μg/mL). (d, e) Kanamycin fluorescence imaging and intensity assay of untreated or 4F-indole-treated P. aeruginosa PAO1. Error bars show the standard deviation of three replicates. A *t* test was performed. **, *P < *0.01. DMSO, dimethyl sulfoxide; CFDA, 5(6)-carboxyfluorescein diacetate; OD_600_, optical density at 600 nm. KAN, kanamycin.

In this study, we found that halogenated indoles, including 4F-indole, 4Cl-indole, 4Br-indole, 7F-indole, and 5I-indole, could reactivate the aminoglycoside antibiotics against P. aeruginosa PAO1 (Fig. 1a; Fig. S1). Halogenated indoles synergized with antibiotics to inhibit the growth of P. aeruginosa PAO1 more effectively, compared with indole (Fig. 1a). We selected 4F-indole as a representative for further study. 4F-Indole was combined with aminoglycoside antibiotics, such as gentamicin, kanamycin, tobramycin, amikacin, neomycin, ribosomalin sulfate, and cisomicin, for the analysis of MIC and MBC. The results reveal that the combination of 4F-indole with antibiotics reduces the MICs (2- to 4-fold) and MBC (2- to 16-fold) compared with antibiotics individually (Table 1). The results suggest that 4F-indole could enhance the effectiveness of traditional antibiotics. The growth curves of P. aeruginosa PAO1 showed that 4F-indole with low concentrations (<0.5 mM) had no significant difference in the growth of P. aeruginosa PAO1 (Fig. 1b; Fig. S2). In contrast, the growth of P. aeruginosa PAO1 was inhibited by the combination of 4F-indole and kanamycin (50 μg/mL), and the difference in growth inhibition between 0.5 and 0.6 mM 4F-indole combined with kanamycin was not significant. We chose a more effective and less toxic concentration (0.5 mM 4F-indole) for further studies (Fig. 1c; Fig. S2). By using fluorescently labeled kanamycin (kanamycin–5(6)-carboxyfluorescein diacetate [CFDA]) to detect its intracellular content, we showed that 4F-indole significantly increased the intracellular content of kanamycin-CFDA in P. aeruginosa PAO1 (Fig. 1d and e). Therefore, we speculate that the 4F-indole-induced inhibition on antibiotic resistance could be mediated by the uptake or efflux of antibiotics.

**TABLE 1:** MIC and MBC of P. aeruginosa PAO1 under antibiotic treatment (with or without 4F-indole)

Aminoglycosides	MIC (μg/mL)		MBC (μg/mL)	
	Antibiotic alone	With 4F-indole	Antibiotic alone	With 4F-indole
Gentamicin	1	0.25	4	2
Kanamycin	8	2	64	16
Tobramycin	1	0.25	4	1
Amikacin	1	0.25	2	0.5
Neomycin	2	0.5	32	2
Ribostamycin	320	160	1,280	640
Sisomicin	0.5	0.25	2	1

### PmrA/PmrB regulates the expression of the MexXY-OprM efflux pump.

**FIG 2:**
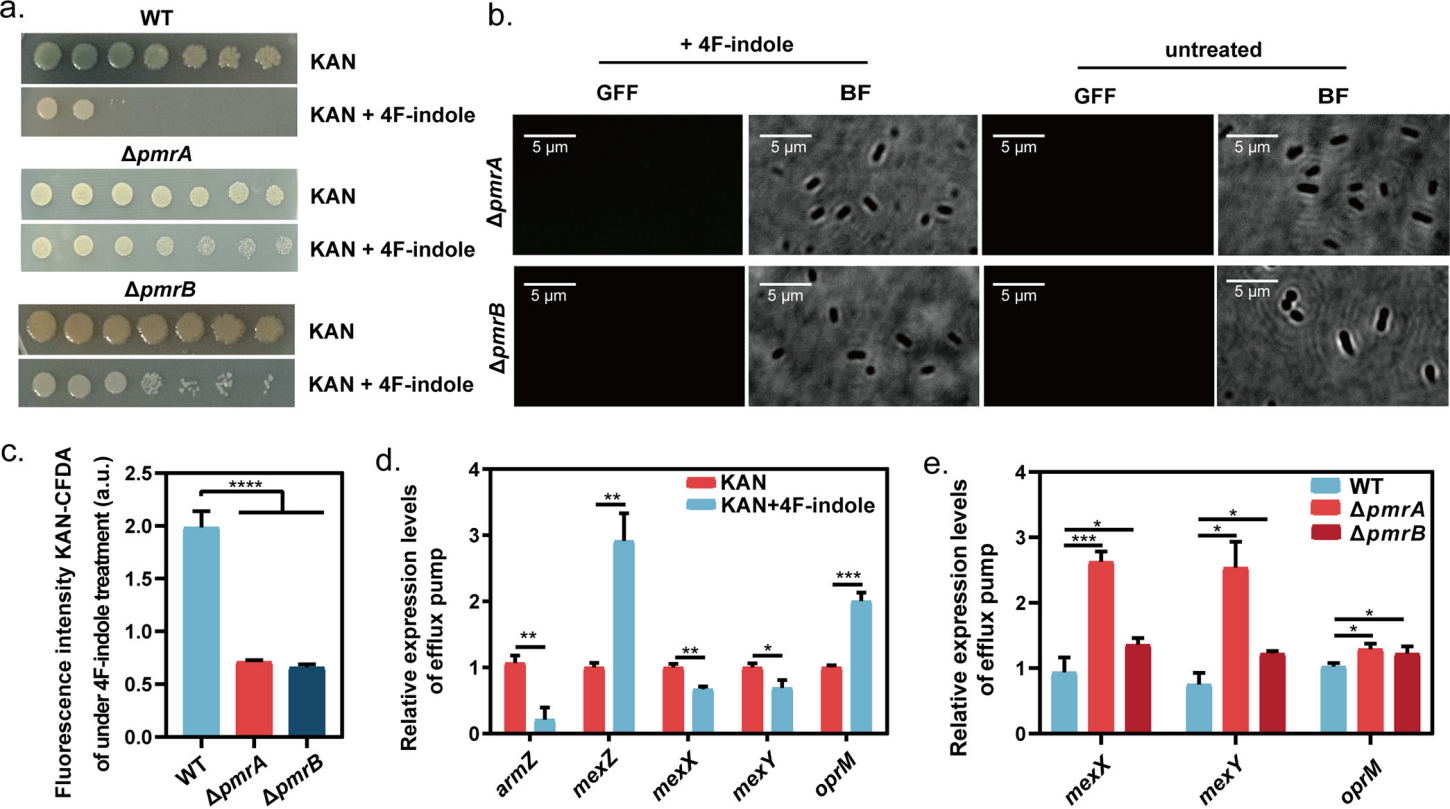
4F-Indole inhibits the expression of MexXY-OprM efflux pump through PmrA/PmrB. (a) Efficiencies of colony formation of P. aeruginosa PAO1 wild-type strain, Δ*pmrA*, and Δ*pmrB* in the presence of 4F-indole and kanamycin. (b, c) Kanamycin fluorescence image and intensity assay for Δ*pmrA* and Δ*pmrB* under 4F-indole treatment. (d, e) Real-time PCR assays of the relative expression levels of MexXY-OprM efflux pump in P. aeruginosa PAO1 wild-type strain, Δ*pmrA*, and Δ*pmrB*. Error bars show the standard deviation of three replicates. A *t* test was performed. *, *P < *0.05; **, *P < *0.01; ***, *P < *0.001; ****, *P* < 0.0001. GFP, green fluorescent protein. KAN, Kanamycin; DFF, Green fluorescent field; BF, Bright field.

P. aeruginosa has multiple TCSs to regulate virulence and antibiotic resistance (7). Based on analysis of the P. aeruginosa PAO1 genome, we focused on PmrA/PmrB that is homologous with QseB/QseC (Fig. S3 and S4). QseB/QseC was reported to sense indole and to regulate indole-induced antibiotic resistance inhibition (31, 32). Therefore, we speculate that PmrA/PmrB might be involved in the 4F-indole resensitization of P. aeruginosa PAO1 to kanamycin. We constructed Δ*pmrA* and Δ*pmrB* mutant strains to verify the speculation. The Δ*pmrA* and Δ*pmrB* mutant strains largely restored growth under 4F-indole and kanamycin combination conditions, compared with that of the wild-type strain (Fig. 2a), suggesting that PmrA/PmrB plays a key role in the recovery of kanamycin susceptibility. Moreover, the lack of *pmrA* or *pmrB* caused the ineffective 4F-indole-mediated regulation on the concentration of intracellular kanamycin-CFDA (Fig. 2b). Under 4F-indole treatment, the fluorescence intensity of Δ*pmrA* and Δ*pmrB* was significantly lower than that of the wild-type strain (Fig. 2c). Real-time quantitative reverse transcription-PCR (RT-qPCR) results showed that 4F-indole induced downregulation of *armZ*, *mexX*, and *mexY* and upregulation of *mexZ* (Fig. 2d). MexZ is the regulator to inhibit the expression of *mexXY* encoding MexXY, an important component of MexXY-OprM antibiotic efflux pump, and ArmZ is the inhibitory regulator of MexZ. Therefore, we speculate that 4F-indole might induce the inhibition of MexXY-OprM, causing high intracellular concentrations of kanamycin by downregulating *armZ* expression. Under 4F-indole treatment, the expression of *mexX*, *mexY*, and *oprM* was upregulated by the absence of *pmrA* or *pmrB* (Fig. 2e). The above results suggest that 4F-indole increases intracellular content of antibiotics through PmrA/PmrB, resulting in the inhibition of resistance of P. aeruginosa PAO1 against antibiotics, and PmrA/PmrB-regulated MexXY-OprM might also be involved in this mechanism.

### 4F-Indole inhibits the biosynthesis of multiple virulence factors.

**FIG 3:**
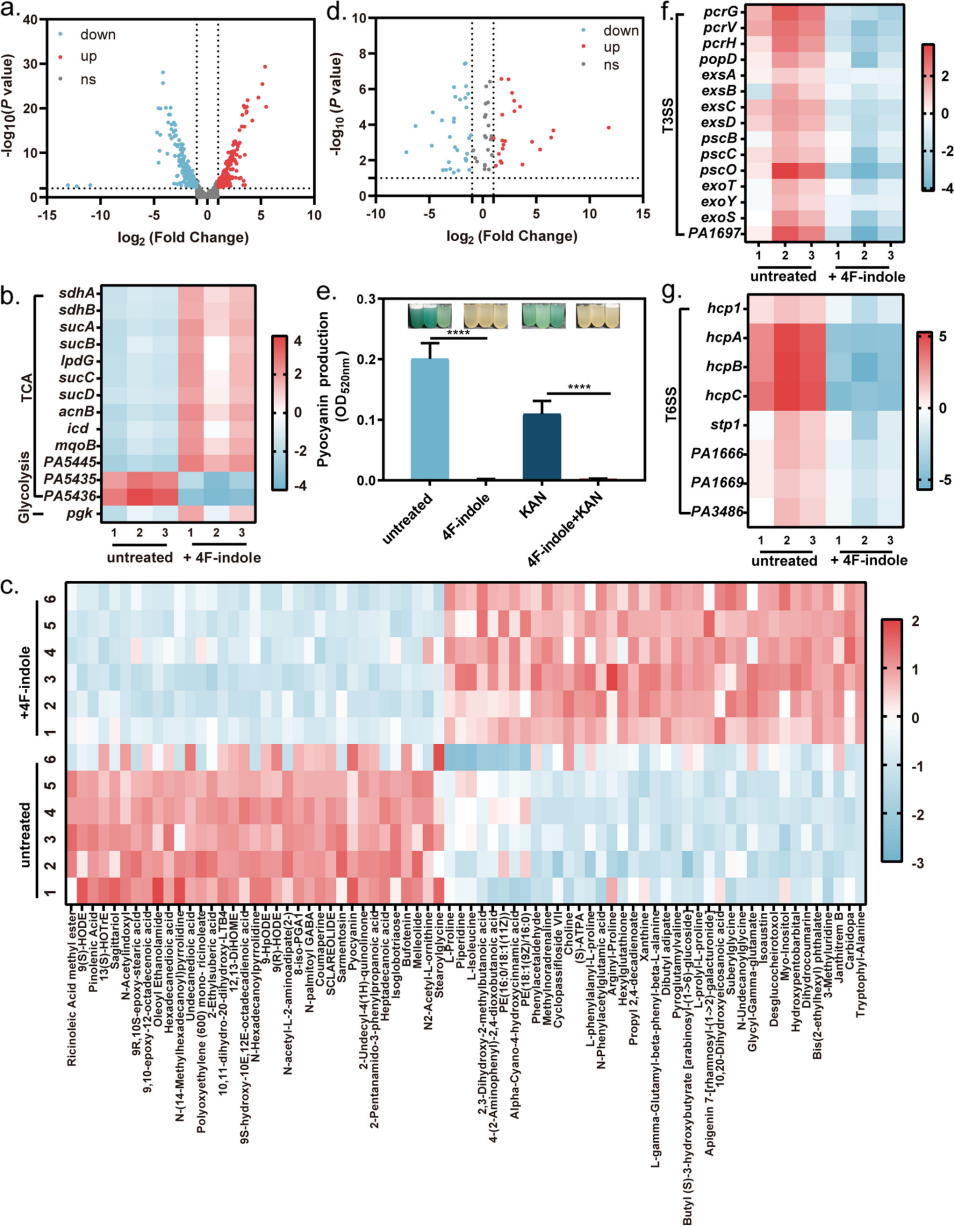
4F-Indole induced significant changes in P. aeruginosa PAO1 at the transcriptional and metabolic levels. (a) Volcano plots of differential genes and metabolites. (b) The heat map of differential genes in the citric acid cycle and glycolysis metabolic pathways. (c) The heat map of differential metabolites. (d) Volcano plots of differential genes and metabolites. (e) The pyocyanin assays of P. aeruginosa PAO1 treated by kanamycin and 4F-indole alone or in combination. (f, g) Heat maps of differential genes in T3SS and T6SS metabolic pathways. Error bars show the standard deviation of three replicates. A *t* test was performed. ****, *P* < 0.0001. TCA, Tricarboxylic acid; T6SS, type VI secretion system. T3SS, type Ш secretion system.

**FIG 4:**
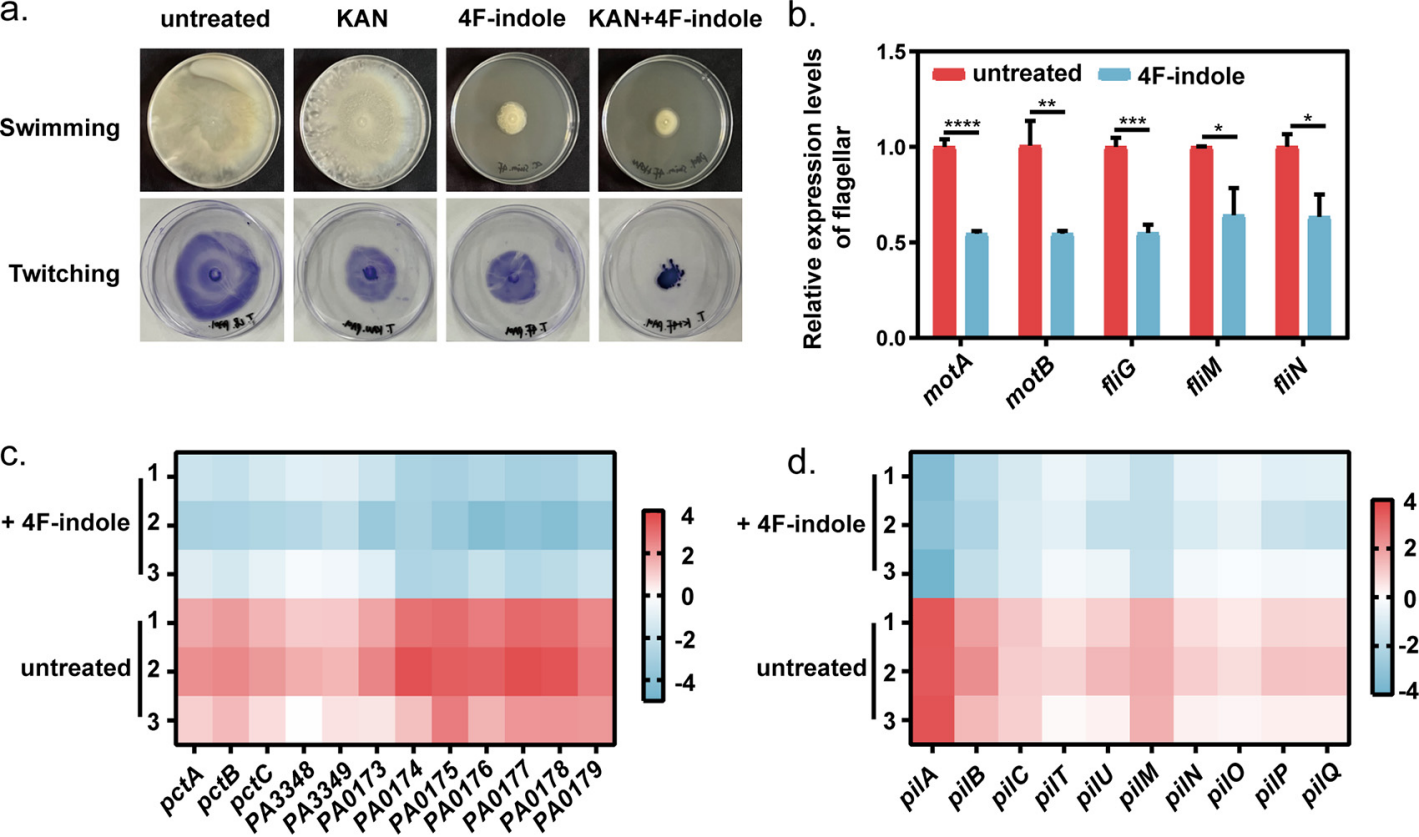
4F-Indole inhibits the motility of P. aeruginosa PAO1. (a) Swimming and twitching motility assays of P. aeruginosa PAO1 treated by kanamycin and 4F-indole alone or in combination. (b) Real-time PCR assays for relative expression levels of key genes of flagellar motor. (c, d) Heat map of differential genes in chemotactic proteins and type IV pili metabolic pathways. Error bars show the standard deviation of three replicates. A *t* test was performed. *, *P < *0.05; **, *P < *0.01; ***, *P < *0.001; ****, *P* < 0.0001. KAN, kanamycin.

Combined transcriptomics and metabolomics were used to analyze the effect of 4F-indole-mediated resistance reduction on the physiological response of P. aeruginosa PAO1. 4F-Indole treatment led to the identification of 260/244 (up/down) differentially expressed genes (DEGs) in P. aeruginosa PAO1 (fold change [FC] > 2, *P < *0.05) (Fig. 3a). The DEGs were significantly enriched in several metabolic pathways, such as carbon fixation, citric acid cycle, glycolysis (Fig. 3b), amino acid biosynthesis and metabolism (Fig. 4c and S5a), ATP-binding cassette (ABC) transporters (Fig. S5d), lipid metabolism (Fig. S5e), and pyocyanin synthesis (Fig. S5f). The principal-component analysis (PCA) scoring plots showed overall metabolic differences between 4F-indole-treated and untreated P. aeruginosa PAO1 (Fig. S6). Metabolomic results showed that a total of 75 significantly different metabolites (Variable important in projection, VIP > 1, *P < *0.05), with 40/35 (up/down) identified (Fig. 3c and d). They are distributed in ABC transporters, aminoacyl-tRNA biosynthesis, biosynthesis of amino acids, tropane, piperidine, and pyridine alkaloid biosynthesis, tryptophan metabolism, 2-oxocarboxylic acid metabolism, and prodigiosin biosynthesis metabolic pathways (Fig. S7).

We found that 4F-indole had a negative effect on the biosynthesis of several virulence factors. Pyocyanin, a virulence factor of P. aeruginosa PAO1, confers resistance to multiple antibiotics, shortens antibiotic-induced bacterial growth lag time in a dose-dependent manner, and protects bacterial survival during antibiotic treatment (33–35). Our results revealed that 4F-indole completely inhibited the synthesis of pyocyanin (Fig. 3e), which was consistent with the metabolomic results (Fig. 3c). Moreover, transcriptome results show that 4F-indole significantly downregulated several key genes of pyocyanin synthesis, such as *pqsA*-*E*, *phnA*, *phnB*, *mvfR*, *phnA1*, and *phnB1* (Fig. S5f), suggesting that 4F-indole might weaken antibiotic resistance by inhibiting pyocyanin production.

In addition, the transcriptomic results showed significant downregulation of multiple genes in type III secretion systems (T3SSs) (Fig. 3f) and type VI secretion systems (T6SSs) (Fig. 3g). T3SS is associated with bacterial colonization, survival, and replication (36). Secretion of T3SS effectors contributes to bacterial colonization of hosts, survival in adverse environments, and defense against eukaryotic predation (37, 38). Under 4F-indole treatment, key genes of T3SS were downregulated, including component genes (*popD*, *pscB*, and *pscO*) and transcriptional regulators (*exsA*, *exsB*, and *exsC*). T6SS regulates bacterial-host interactions by secreting toxins and participates in biofilm formation (39). For T6SS, the expression of *hcp1*, *hcpA*, *hcpB*, *hcpC*, and *stp1*, which are responsible for structural component genes, was significantly downregulated under 4F-indole treatment (Fig. 3g). The transcript levels of the PldA and Azu, virulence effectors secreted by T6SS, were also downregulated, which implies that the function of T6SS was also affected. These results suggest that 4F-indole is effective in reducing the virulence of P. aeruginosa PAO1.

### 4F-Indole inhibits the swimming and twitching motility of P. aeruginosa PAO1.

The motility mediated by flagella and type IV pili is an important mechanism of antibiotic resistance in P. aeruginosa PAO1 (40). 4F-Indole showed a significant inhibitory effect on the motility of P. aeruginosa PAO1. The results of the motility plate showed that 4F-indole inhibited the swimming motility of P. aeruginosa PAO1 (Fig. 4a). It was consistent with the RT-qPCR results that 4F-indole suppressed the transcript levels of flagellar motor critical genes *motA*, *motB*, and *fliG* (Fig. 4b). Transcript levels of these chemotaxis-related genes were downregulated under 4F-indole treatment, such as Che (*PA3348* to *PA3349*), Che2 (*PA0173* to *PA0179*), *pctA*, *pctB*, and *pctC* (Fig. 4c). We speculated that 4F-indole could cause downregulation of chemotactic proteins expression and reduced flagellar synthesis, thus inhibiting the swimming motility of P. aeruginosa PAO1.

The motility plate assay shows that 4F-indole and kanamycin treatment alone inhibited the twitching motility of P. aeruginosa PAO1. Moreover, the combination of the two agents revealed a stronger synergistic inhibition effect (Fig. 4a). P. aeruginosa PAO1 utilizes type IV pili for twitching motility on the surface of the adherent (41). Under 4F-indole treatment, the transcript levels of key genes of type IV pili were significantly downregulated, including the major component protein subunit PilA, platform protein PilC, ATPase motor protein PilB, retraction motor PilT, parter ATPase PilU, secretory protein PilQ, and alignment protein PilM, PilN, PilO, PilP, etc. (Fig. 4d). We speculated that 4F-indole might inhibit the twitching motility by controlling the key proteins of bacterial T4P synthesis, extension, and contraction in P. aeruginosa PAO1. In summary, 4F-indole could enhance the susceptibility of P. aeruginosa PAO1 to aminoglycoside antibiotics by acting on multiple aspects of drug efflux pump, virulence factors, and bacterial motility.

## DISCUSSION

**FIG 5:**
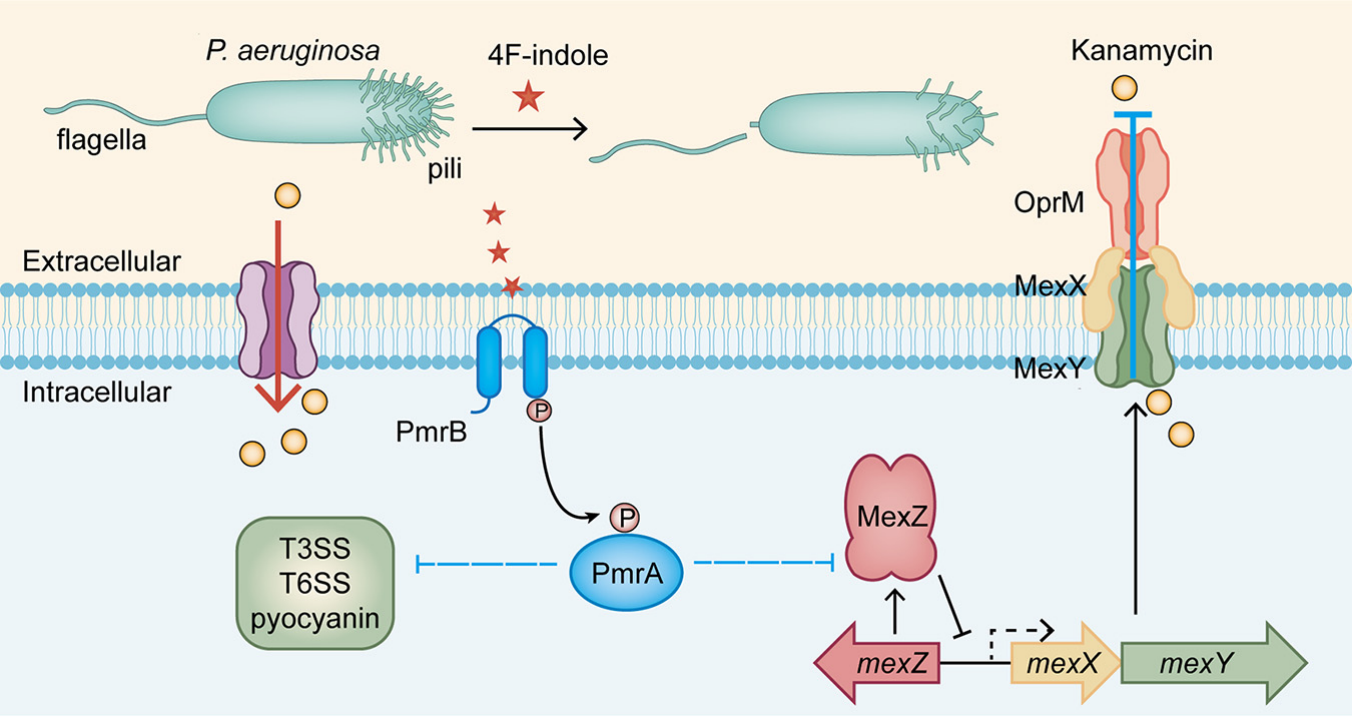
Physiological effects caused by 4F-indole on P. aeruginosa PAO1 and mechanisms to enhance its susceptibility to aminoglycoside antibiotics. T3SS, type III secretion system; T6SS, type VI secretion system.

In this study, we showed that 4F-indole increases the susceptibility of P. aeruginosa PAO1 to aminoglycoside antibiotics. To investigate this mechanism, we explored the regulatory effect of 4F-indole on antibiotic resistance of P. aeruginosa PAO1. We found that PmrA/PmrB plays a critical role in the regulation of antibiotic sensitivity by 4F-indole, which reduces the expression of MexXY-OprM efflux pump and might lead to intracellular kanamycin accumulation (Fig. 5). Notably, we verified the 4F-indole-mediated promotion on intracellular content of kanamycin-CFDA and confirmed the important role of PmrA/PmrB in this regulatory process. However, our evidence is not enough to reveal the effect of CFDA in this process. This issue will be resolved in our future studies. Moreover, 4F-indole inhibited the expression of flagella and type IV pili and then reduced the swimming and twitching motility of P. aeruginosa PAO1. Bacterial motility, such as swimming and twitching, is one of the social activities of bacteria and is essential to lifestyle transition and antibiotic resistance (42, 43). 4F-Indole also inhibited the biosynthesis of several virulence factors, including pyocyanin and effectors of T3SS and T6SS. It has been shown that the expression of virulence is associated with resistance (44).

Many Gram-negative and Gram-positive bacteria could synthesize indoles, which are effective in inhibiting quorum sensing and reducing the virulence of pathogenic bacterial (45). It is reported that although this signal is effective in reducing the virulence of P. aeruginosa, the antibiotic resistance was enhanced (46), which suggests that indole has some limitations on antibacterial treatment. So, are indole analogues with tiny structural differences more promising in antimicrobial therapy than indoles? The halogenated indole was subsequently found to reduce bacterial virulence as well. For example, 7F-indole was effective in reducing biofilm formation and inhibiting the biosynthesis of virulence factors (27). In contrast, our study found that 4F-indole had a broader effect on inhibiting the synthesis of virulence factors and effectively reducing antibiotic resistance, and 4F-indole also had the same effect on reducing resistance to aminoglycoside antibiotics in clinically multidrug-resistant P. aeruginosa, which makes the combination of 4F-indole with traditional antibiotics have potential clinical value (Tables S1 and S2). Interestingly, while 4F-indole does not inhibit biofilm formation (Fig. S8), it appears to directly inhibit the secretion system and the expression of virulence factors. The key gene functions and internal regulatory mechanisms behind this physiological phenomenon need to be further studied.

4F-Indole influenced several phenotypes of P. aeruginosa. In order to deeply reveal the mechanism, we performed global metabolomics analysis. Regulation on bacterial metabolism is critical to antibiotic therapy, and altering bacterial physiology through external conditions can affect antibiotic susceptibility (47, 48). For example, the killing of persister cells by aminoglycoside antibiotics can be enhanced by stimulation with glucose and pyruvate (47). Notably, 4F-indole induced changes in the transcriptome of central carbon metabolism (Fig. 3b), especially the key genes in tricarboxylic acid cycle, with 11/2 (up/down) DEGs, including succinate dehydrogenase (*sdhA* and *sdhB*), 2-oxoglutarate dehydrogenase (*sucA*), succinyltransferase (*sucB*), and succinyl-CoA synthase (*sucC* and *sucD*) (Fig. 3b). Moreover, considering that proton motive force (PMF) is reported to affect the sensitivity of aminoglycoside antibiotics (49), we hypothesize that 4F-indole may enhance bacterial killing by facilitating the Tricarboxylic acid (TCA) cycle in bacteria and providing the PMF necessary for kanamycin uptake. It has been shown that fumarate activates cellular respiration by stimulating the TCA cycle and generates PMF to promote tobramycin uptake by P. aeruginosa (49). 4F-Indole promoted the overexpression of succinate dehydrogenase, which induced the conversion of succinate to fumarate (Fig. 3b), and this result also supported the above speculation.

The development and spread of antibiotic resistance in bacteria are a general threat to humans and animals, and the difficulty of discovering new antibiotics is increasing (50). Current treatment options to address infections caused by resistant P. aeruginosa are mainly a combination of multiple antibiotics (51). The application of antibiotic adjuvants to enhance the effectiveness of existing antibiotics is one of the rapid and effective strategies to address antibiotic resistant bacterial infections (52). This study concluded that 4F-indole has potential as an aminoglycoside antibiotic adjuvant, providing a novel perspective for the treatment of infections caused by P. aeruginosa PAO1 with traditional antibiotics. In addition, it provides a theoretical basis for the discovery of novel indole derivatives for the treatment of resistant bacterial infections.

## MATERIALS AND METHODS

### Colony formation efficiency assay.

After overnight incubation at 37°C, the passaged cultures of P. aeruginosa PAO1 were inoculated into Luria-Bertani (LB) medium at 1% inoculum, incubated at 37°C and 170 rpm until the optical density at 600 nm (OD_600_) value to 0.6, serially diluted with LB medium in a gradient to10^−1^, 10^−2^, 10^−3^, 10^−4^, 10^−5^, 10^−6^, and 10^−7^ of the original culture solution. A total of 3 μL of the original solution and diluted culture solution were pipetted onto solid plates containing kanamycin (50 μg/mL) with or without indole compounds (0.5 mM). The indole analogues included indole, 4F-indole, 4Cl-indole, 4Br-indole, 7F-indole, and 5I-indole. All experiments were repeated three times independently.

### Growth curves assay.

The cultures of P. aeruginosa PAO1 were grown overnight at 37°C and inoculated into LB liquid medium containing 4F-indole, kanamycin, or both at 1% inoculum, respectively, and incubated at 37°C and 170 rpm. The OD_600_ was measured every 1 h by using spectrometer. All experiments were repeated three times independently.

### MIC and MBC assay.

The MIC of antibiotics combined with 4F-indole for P. aeruginosa PAO1 was determined using a 2-fold dilution technique in 96-well microtiter plates, as described by the Clinical and Laboratory Standards Institute (CLSI) guidelines. In this assay, P. aeruginosa PAO1 was grown in Mueller-Hinton (MH) broth and prepared as a bacterial suspension for use. Antibiotics with an original concentration of 1,024 μg/mL were used to make 2-fold dilutions. The original concentration of ribostamycin before dilution was 10.24 mg/mL because of its high MIC value against P. aeruginosa PAO1. First, 50 μL MH (or MH containing 0.5 mM 4F-indole) was added to each well of a 96-well plate, followed by 50 μL of antibiotic (1,024 μg/mL) in the first well, and 2-fold gradient dilution was performed. The cultured bacterial suspension was adjusted to a turbidity of 55 and then diluted 100-fold, 50 μL of bacterial suspension was added to each well (to obtain a final concentration of approximately 5 × 10^5^ CFU/mL), and the results were recorded after incubation at 37°C for 18 h. The measurements were performed in triplicate.

The broth dilution method to determine the MIC of antibiotics on bacteria based on the naked eye was no bacterial growth in each tube. A total of 100 μL of test bacterial solution was transferred to the agar dishes without antibiotics. After overnight incubation, the number of colonies growing on each plate <0.1% of the inoculum amount of the corresponding broth tube of the minimum drug concentration (i.e., <5 colonies corresponding to the minimum drug concentration), that is, the MBC. All of the experiments were repeated three times independently.

### Transcriptomic and metabolomic analysis.

Transcriptional profiling of P. aeruginosa PAO1 (in the absence or presence of 0.5 mM 4F-indole) was carried out by the Biozeron Company, Shanghai, China (PRJNA625005). Total RNA of P. aeruginosa PAO1 was extracted with TRIzol reagent (Invitrogen), and genomic DNA was removed using DNase I (TaKara). Then RNA quality was quantified using a Bioanalyzer 2100 (Agilent) and NanoDrop 2000. RNA-sequencing strand-specific libraries were prepared by a TruSeq RNA library preparation kit for Illumina. Library sequencing was performed on the Illumina HiSeq platform. EdgeR was used for differential gene expression analysis (http://bioconductor.org/packages/-release/bioc/html/edgeR.html). Clean reads were aligned to the reference genome using Rockhopper (http://cs.wellesley.edu/~btjaden/Rockhopper/). Gene Ontology (GO) functional enrichment and Kyoto Encyclopedia of Genes and Genomes (KEGG) pathway analysis were carried out by Goatools (https://github.com/tanghaibao/Goatools) and KOBAS, respectively (http://kobas.cbi.pku.edu.cn/home.do). Differences greater than 2-fold with *P < *0.05 were regarded as significant differences.

### Kanamycin fluorescence assay.

Fluorescence labeling of kanamycin (C_18_H_38_N_4_O_15_S; MW = 582.58) was added to anhydrous dimethylformamide (DMF), and then triethylamine was added to the reaction system. 5(6)-Carboxyfluorescein diacetate and succinimidyl ester (CFDA-SE) was dissolved in DMF and added to the kanamycin solution for a 4 h reaction. Thin-layer chromatography (TLC) detection and high-performance liquid chromatography (HPLC) purification were carried out. Mass spectrometry (MS) was used to verify the chemical structure of kanamycin-CFDA. For kanamycin-CFDA staining, the final concentration is 50 μg/mL. The cells were incubated in dark environment with slow shaking at 37°C for 5 h. The cells were collected and washed three times with 0.85% NaCl solution and then observed under a fluorescence microscope. The fluorescence intensity was measured by Microplate Reader Synergy H1 with λ_ex_ of 488 nm and λ_em_ of 525 nm. All experiments were repeated three times independently.

### Construction of deletion mutants in strain P. aeruginosa PAO1.

The mutants in this study were obtained using Red/ET homologous recombination (53), and the *pmrA* gene was used as an example to elucidate the method. The gentamicin resistance gene was used to replace the *pmrA* gene in the P. aeruginosa PAO1-red (PAO1 containing Red/ET recombinant system plasmid) genome. The PCR product contained a 100-bp sequence homologous to the *pmrA* gene at the 5′ end and a 20-bp sequence homologous to the gentamicin resistance gene at the 3′ end; it was electroporated by eporator (1.37 kV, 4 ms) into a P. aeruginosa PAO1-red-expressing Red/ET recombineering system. Mutations in the *pmrA* gene were verified using primers upstream and downstream of the *pmrA* gene (*pmrA*-check1 and *pmrA*-check2).

### RT-qPCR.

P. aeruginosa PAO1 strains were grown in LB medium and harvested until grown to log phase (the value of OD_600_ is 0.4 to 0.6), and then the organisms were collected with or without 0.5 mM 4F-indole treatment for 30 min. RNA was extracted using the E.Z.N.A.^R^ bacterial RNA kit (R6950-01, Omega) following the manufacturer’s instructions. Genomic DNA was removed by its DNA eraser kit, and the total cDNA was synthesized by the HiScript II reverse transcriptase (Vazyme). RT-qPCR was performed by using the SYBR green real-time PCR Master Mix and the Step-One-Plus real-time PCR system (ABI). For calculation of the relative expression levels of tested genes, the 16S rRNA gene was used as the reference gene. All experiments were repeated three times independently.

### Swimming and twitching motility assay.

Swimming and twitching motility of P. aeruginosa PAO1 was detected with LB semisolid plates of 0.5% and 1% agar, respectively, P. aeruginosa PAO1 was pierced into the interior of LB 0.3% agar plates (no touching the bottom) with a toothpick, and the plates were incubated at 37°C for 20 h and then observed for swimming movement area. For the twitching motility assay, P. aeruginosa PAO1 has pierced into the bottom of LB 1% agar plates with a toothpick, and the plates were incubated at 37°C for 48 h. Then, the agar was carefully removed, and the plastic petri dishes were stained with 1% crystal violet for 20 min. The excess dye was washed away with water, and the purple twitched area was observed. All experiments were repeated three times independently.

### Pyocyanin extraction and determination assay.

P. aeruginosa PAO1 was incubated for 16 to 18 h in 37°C and centrifuged at 8,000 × *g* for 10 min. The supernatant was collected, and chloroform was added in a ratio of 5:3 (vol/vol) for vortex extraction and centrifuged at 13,000 × *g* for 2 min. The chloroform layer was collected. Then 200 μL of 0.2 M hydrochloric acid and vortex mix were added and centrifuged at 8,000 × *g* for 10 min, and 200 μL of the upper layer solution was centrifuged at 8,000 × *g* for 10 min. OD_520_ was measured in a 96-well plate. All experiments were repeated independently at least three times.
