# Supplementary material for: 4F-Indole Enhances the Susceptibility of Pseudomonas aeruginosa to Aminoglycoside Antibiotics
Source: Microbiol Spectr. 2023 Mar 28;11(2):e04519-22. doi: 10.1128/spectrum.04519-22 (PMC10100892; doi:10.1128/spectrum.04519-22)
Supplement: Supplemental file 1 — Supplemental material. Download spectrum.04519-22-s0001.pdf, PDF file, 0.8 MB [file spectrum.04519-22-s0001.pdf]

4F-indole enhances the susceptibility of *Pseudomonas aeruginosa* to aminoglycoside antibiotics

Qin Dou[1], Yuxiang Zhu[1], Chunhui Li[1], Zeran Bian[1], Huihui Song[1], Ruizhen Zhang[1], Yingsong Wang[1], Xile Zhang[1], Yan Wang[1]*

[1] College of Marine Life Sciences, and Institute of Evolution & Marine Biodiversity, Ocean University of China, Qingdao 266003, China

*Correspondence: wangy12@ouc.edu.cn

ORCID: 0000-0003-4632-5786

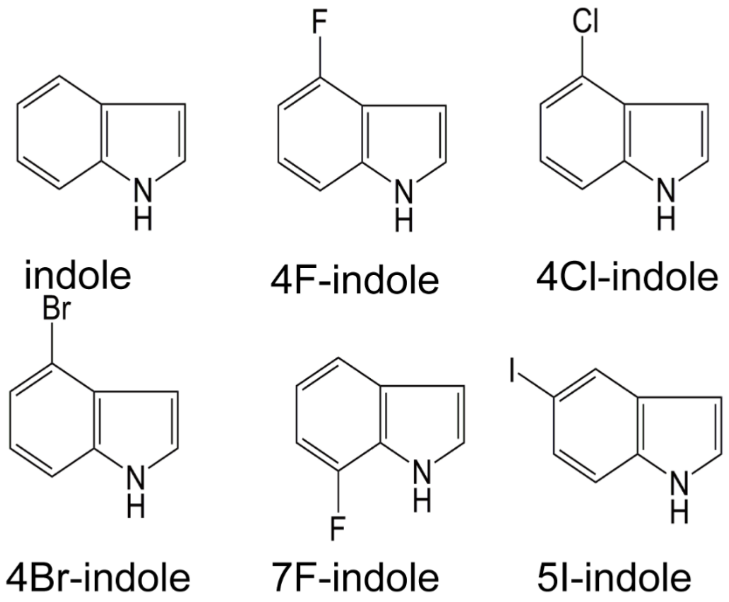

Supplementary figure 1. The structural formula of indole compounds, including indole, 4F-indole, 4Cl-indole, 4Br-indole, 7F-indole, and 5I-indole.

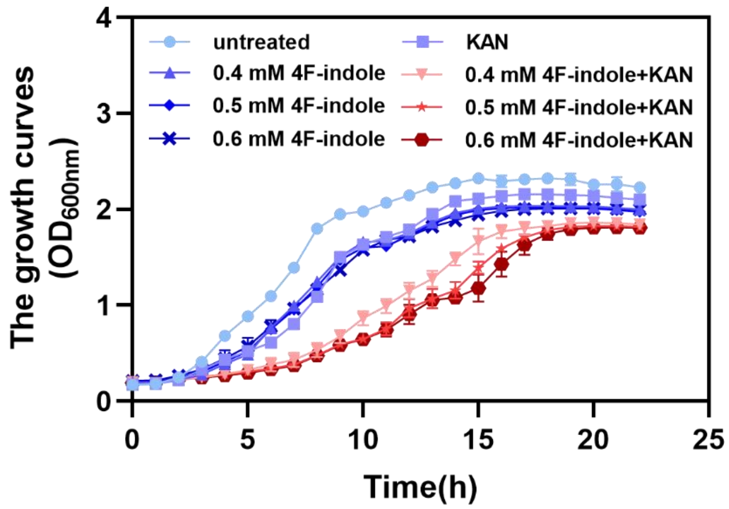

Supplementary figure 2. The growth curves of *P. aeruginosa* PAO1 treated by combinations of 4F-indole (0 mM, 0.4 mM, 0.5 mM, 0.6 mM) with kanamycin (0 μg/mL, 50 μg/mL).

```
CLUSTAL O(1.2.4) multiple sequence alignment


sp|P52076|qseB_E.coli          MRILLIEDDMLIGDGIKTGLSKMGFSVDWFTQGRQGKEALYSAPYDAVILDLTLPGMDGR    60
sp|Q9HV32|pmrA_Pseudomonas     MRILLAEDDLLLGDGIRAGLRLEGDTVEWVTDGVAAENALVTDEFDLLVLDIGLPRRSGL    60
                               ***** ***:*:****::**   * :*:*.*:*  .::** :  :* ::**: **  .* 

sp|P52076|qseB_E.coli          DILREWREKGQREPVLILTARDALAERVEGLRLGADDYLCKPFALIEVAARLEALMRRTN    120
sp|Q9HV32|pmrA_Pseudomonas     DILRNLRHQGLLTPVLLLTARDKVADRVAGLDSGADDYLTKPFDLDELQARVRALTRRTT    120
                               ****: *.:*   ***:***** :*:** **  ****** *** * *: **:.** ***.

sp|P52076|qseB_E.coli          GQASNELRHGNVMLDPGKRIATLAGEPLTLKPKEFALLELLMRNAGRVLSRKLIEEKLYT    180
sp|Q9HV32|pmrA_Pseudomonas     GRALPQLVHGELRLDPATHQVTLSGQAVELAPREYALLRLLLENSGKVLSRNQLEQSLYG    180
                               *:*  :* **:: ***..: .**:*: : * *:*:***.**:.*:*:****: :*:.** 

sp|P52076|qseB_E.coli          WDEEVTSNAVEVHVHHLRRKLGSDFIRTVHGIGYTLGEK--    219
sp|Q9HV32|pmrA_Pseudomonas     WSGDVESNAIEVHVHHLRRKLGNQLIRTVRGIGYGIDQPAP    221
                               *. :* ***:************.::****:**** :.:   
```

Supplementary figure 3. Relative comparison of protein sequences between PmrA/B and QseB/C. The sequence identity of PmrA and QseB is 53.88%.

```
CLUSTAL O(1.2.4) multiple sequence alignment

sp|qseC_Escherichia                 MKFTQRLSLRVRLTLIFLILASVTWLLSSFVAWKQTTDNVDELFDTQLMLFAKRLS-TLD        59
sp|pmrB_Pseudomonas_aeruginosa      MSRAAVPSVRRRLLVNLLVGFVLCWLSVAALTYHLSLKQVNRLFDDDMVDFGEAALRLLD        60
                                    *. :   *:* ** : :*:   : **  : :::: : .:*:.*** ::: *.:     **

sp|qseC_Escherichia                 LNEINAADRMAQTPNRLKHG---------HVDDDALTFAIFTHDGRMVLNDGDNGEDIPY        110
sp|pmrB_Pseudomonas_aeruginosa      LATEDQAGEDGSITEIIERSREAIQGLPLLRRESALGYALWRD-GQPLLSS----LNLPP        115
                                    *   : *.. ..  : :::.            :.** :*:: . *: :*..     ::*

sp|qseC_Escherichia                 SYQREGFADGQLVGEDDPWRFVWMTSPDGKYRIVVGQEWEYREDMALAIVAGQLIPWLVA        170
sp|pmrB_Pseudomonas_aeruginosa      EITAQGPGFSTVEAQGTHWRVLQLNI--DGFQIWISENLIYRQHTMNLLLFYSLFPLLLA        173
                                    .   :* . . : .:.  **.: :.   . ::* :.::  **:.    ::  .*:* *:*

sp|qseC_Escherichia                 LPIMLIIMMVLLGRELAPLNKLALALRMRDPDSEKPLNATGVPSEVRPLVESLNQLFART        230
sp|pmrB_Pseudomonas_aeruginosa      LPLLGGLVWFGVARGLAPLREVQAEVQQRSARHLQPIAVEAVPLEIRGLIDELNLLLERL        233
                                    **::  :: . :.* ****.::   :: *.    :*: . .** *:* *::.** *: *

sp|qseC_Escherichia                 HAMMVRERRFTSDAAHELRSPLTALKVQTEVAQLSDDDPQARKKALLQLHSGIDRATRLV        290
sp|pmrB_Pseudomonas_aeruginosa      RTALEAERRLTSDAAHEIRTPLASLRTHAQVA-LRSEDPKAHARGLLQVSRSVERISTLM        292
                                    :: :  ***:*******:*:**::*:.:::** * .:**:*: :.***:  .::* : *:

sp|qseC_Escherichia                 DQLLTLSRLDSLDNLQDVAEIPLEDLLQSSVMDIYHTAQQAKIDVRLTLNAHSIKRTGQP        350
sp|pmrB_Pseudomonas_aeruginosa      EQILLLARLDGDALLEQFHPVNLATLAEDVLSELAR--QAIDKDIELSLHQETVYVMGID        350
                                    :*:* *:***.   *::.  : *  * :. : :: :  *  . *:.*:*: .::   *

sp|qseC_Escherichia                 LLLSLLVRNLLDNAVRYSPQGSVVDVTLNA----DNFIVRDNGPGVTPEALARIGERFYR        406
sp|pmrB_Pseudomonas_aeruginosa      LWLKAMVGNLVGNALRYTPAGGQVEIRVENRAQHAVLRVRDNGPGVALEEQQAIFTRFYR        410
                                    * *. :* **:.**:**:* *. *:: ::       : ********: *    *  ****

sp|qseC_Escherichia                 PPGQ-TATGSGLGLSIVQRIAKLHGMNVEFGNAEQG-GFEAKVSW--------------        449
sp|pmrB_Pseudomonas_aeruginosa      SPATSSGEGSGLGLPIVKRIVELHFGSIGLGKGLEGKGLEVQVFLPKTQPDATRPPARGP        470
                                     *.  :. ****** **:**.:**  .: :*:. :* *:*.:*

sp|qseC_Escherichia                 -------     449
sp|pmrB_Pseudomonas_aeruginosa      DSGRSHI     477
```

Supplementary figure 4. Relative comparison of protein sequences between PmrA/B and QseB/C. The sequence identity of PmrB and QseC is 30.53%.

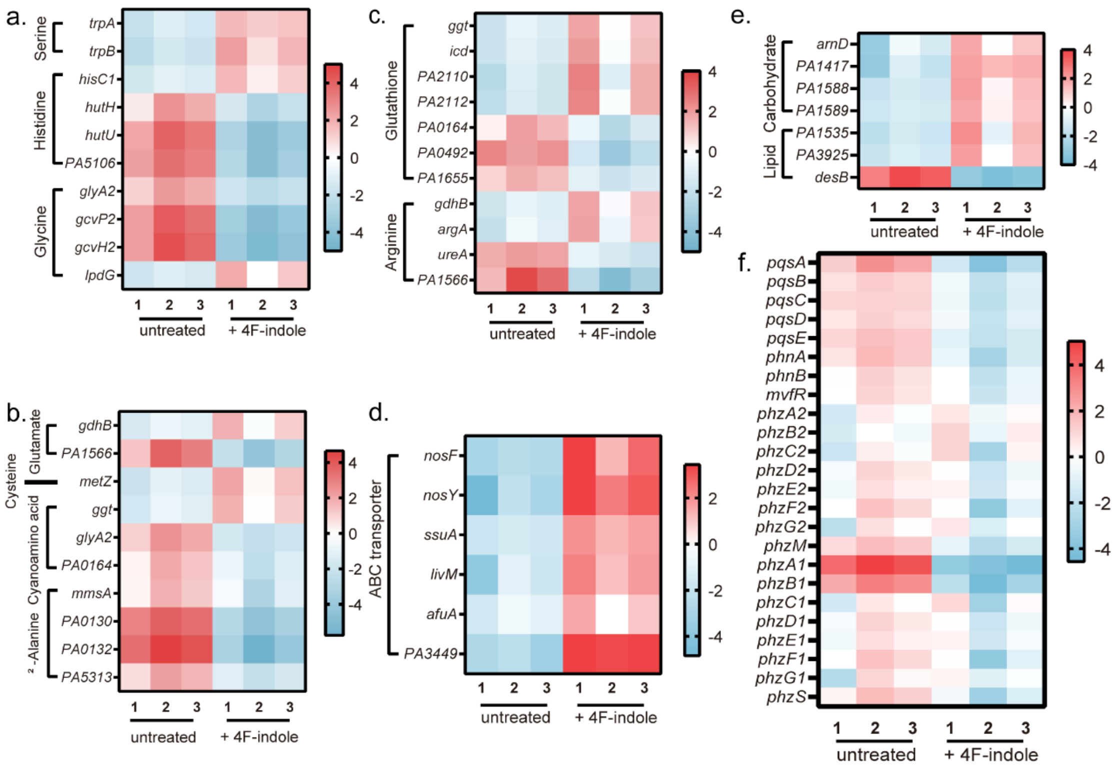

Supplementary figure 5. The heat map of differential genes in diverse metabolic pathways. **a-c,** The heatmap of differentially expressed genes for amino acid biosynthesis and metabolism. **d,** The heat map of differential genes in ABC transporters metabolism. **e,** The heatmap of differentially expressed genes for lipid, amino sugar, and nucleotide sugar metabolism (*arnD*), and C5-branched dibasic acid metabolism (*PA1417, PA1588, and PA1589*). **f,** The heat map of genes in pyocyanin biosynthesis pathways.

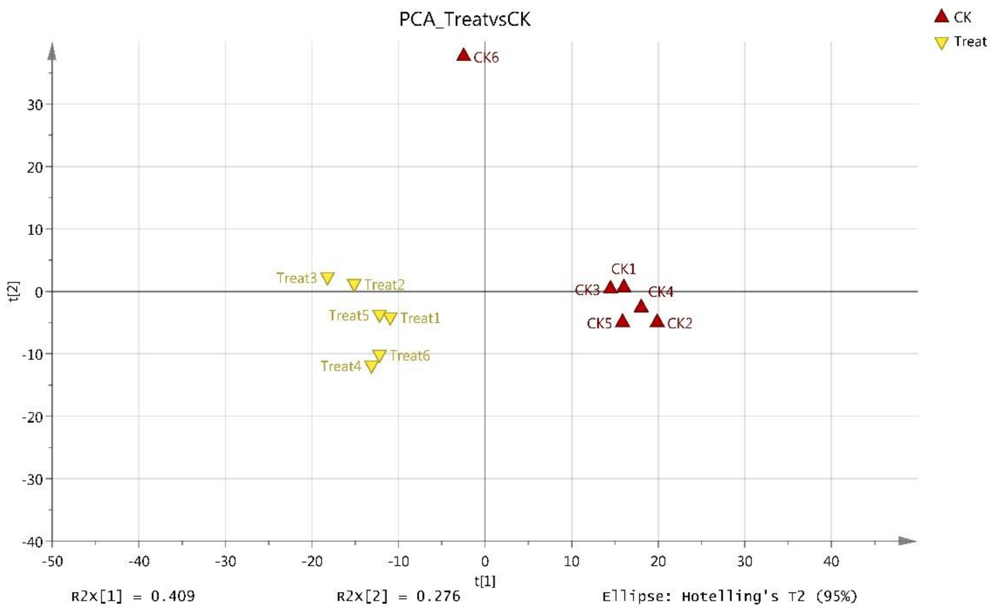

Supplementary figure 6. Principal component analysis (PCA) showed a greater degree of dispersion in the point distributions of 4F-indole treated and untreated samples, indicating a large difference in their metabolomic results.

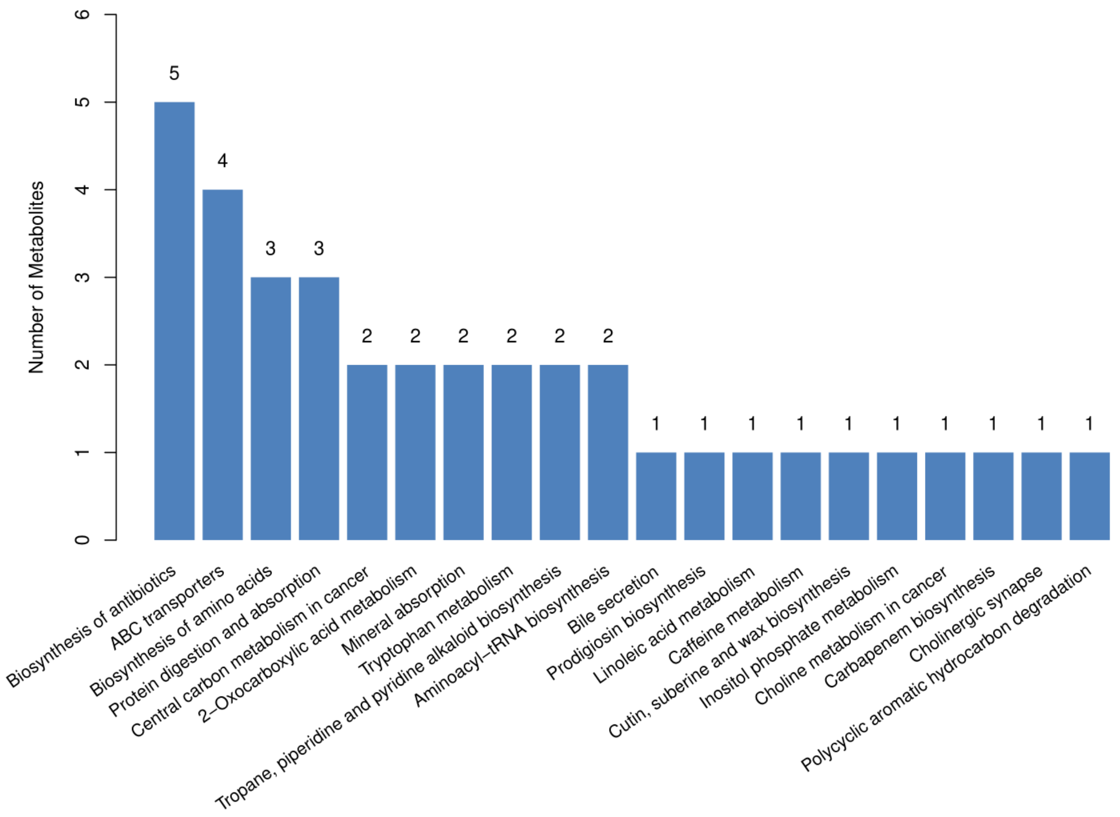

Supplementary figure 7. Metabolomics shows the top 20 pathways that contain the most differential metabolites. From left to right in order of the number of metabolites contained from highest to lowest, with higher columns indicating that the biological pathway is more active in the sample measured.

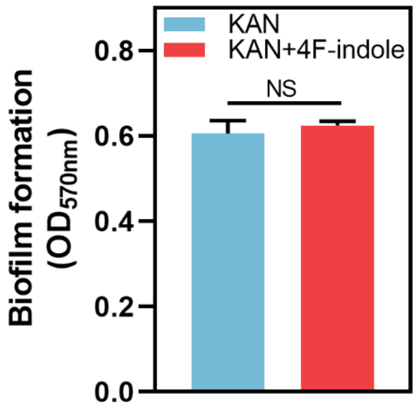

Supplementary figure 8. The biofilm formation assays of *P. aeruginosa* PAO1 treated by kanamycin alone and combination with 4F-indole. Error bars show the standard deviation of three replicates. NS, not significant. All data are mean ± s.e.m.

Supplementary Table 1. The MIC of *P. aeruginosa* under kanamycin treatment (with or without 4F-indole).

Clinical *P. aeruginosa*	MIC (μg/mL)	
	Kanamycin alone	With 4F-indole
*Pa*31786	2	1
*Pa*32034	0.25	0.125
*Pa*21954	0.25	0.125

Supplementary Table 2. The MIC of *Pa*21954 under aminoglycoside antibiotic treatment (with or without 4F-indole).

*Pa*21954	MIC	
	antibiotic alone	With 4F-indole
Gentamicin	0.125	0.0625
Tobramycin	0.125	0.0625
Amikacin	0.5	0.0625

Supplementary Table 3. Bacterial strains and plasmid used in this study.

Strains and plasmid	Relevant characteristics
Strains	
Pseudomonas aeruginosa PAO1	Wild-type
Pseudomonas aeruginosa PAO1 carry pBBR1-Rha-redγ-BAS-Kan plasmid	This strain was used to construct gene deletion mutant strains.
Δ*pmrA*	*pmrA* gene deletion strain
Δ*pmrB*	*pmrB* gene deletion strain
Plasmid	
pBBR1-Rha-redγ-BAS-Kan	The plasmid can express the proteins (Kan^r; $RhaS/R^+$, $Red\gamma^+$, $Red\beta^+$, $Red\alpha^+$, SSB) for gene knockout.

Supplementary Table 4. Primers used in this study.

Primers	Sequences	Purpose
armZ-up	5’- GACAACTACCTGACCCTGCT- 3’	qPCR
armZ-down	5’- TTGTACAGGTTGACCTCGCT- 3’	
mexZ-up	5’- GATCGAGGTACCCGACGAAA- 3’	
mexZ-down	5’- CGTTCGCACTTGAGGTAGAG -3’	
mexX-up	5’- CTGTTCCGCAATCCGCATC -3’	
mexX-down	5’- CCTTTGGGTTGACCACCTTG -3’	
mexY-up	5’- CTCGGTGTTGATCGTGTTCC -3’	
mexY-down	5’- GATGAGGATGGCGTTCTTCG -3’	
oprM-up	5’- GACAACTACCTGACCCTGCT -3’	
oprM-down	5’- TTGTACAGGTTGACCTCGCT -3’	
Δ*pmrA*-up-F	5’- CCGGGGCGAACTGCGGGTCGAATGGCTGATCGTCGCCATCC TCCTGCTGGAGGCCGGCCTGAGCCTGTGGGAATTGTGGAATCA CTGAAACGAGGCTGCCAGCTGAATTACATTCCCAAC -3’	primers for *pmrA* gene deletion
Δ*pmrA*-up-R	5’- AGCCAGCACAGCACGAAGCCGACCAGCAGGTTGACCAGCA GGCGCCGGCGGACGGAGGGGACGGCGGCACGGGACATCGGGG GACTCCGGTAGGCAGTTTCAACTTAAATGTGAAAGTGG -3’	
Δ*pmrA* -yz-F	5’- TCATCGGCAACCCCGAGC -3’	
Δ*pmrA* -yz-R	5’- GCAGACCCTGGATCGCTT -3’	
Δ*pmrB*-up-F	5’- TGCGGCGCAAGCTCGGCAACCAGTTGATCCGCACCGTCCGC GGCATCGGCTACGGCATCGACCAGCCGGCGCCCTGAAAACTGC CTACCGGAGTCCCCCGAGCTGAATTACATTCCCAA -3’	primers for *pmrB* gene deletion
Δ*pmrB* -up-R	5’- TCAGTCCGCTTTTCTTCGCCGCTTCACCGATGTTCATCCGGG TCTCCTGGGAGGTTGCGAAGCCGCTAGCCTATCCCTTTCCCGG CCAATGCGCAGGCTACAACTTAAATGTGAAAGTGG -3’	

Δ*pmrB* -yz-F	5’- GCGATACCGTGGAATGGGTG -3’	
Δ*pmrB* -yz-R	5’- CAACTGGGTTCGTGCCTTCAT -3’	
